# Ceftriaxone-Induced Thrombotic Thrombocytopenic Purpura Treated Successfully With Plasmapheresis and Eculizumab: A Rare Case Report

**DOI:** 10.7759/cureus.48898

**Published:** 2023-11-16

**Authors:** Zaheer A Qureshi, Faryal Altaf, Mikail Khanzada, Aung Thet, Luis Espinosa

**Affiliations:** 1 Internal Medicine, The Frank H. Netter M.D. School of Medicine at Quinnipiac University, Bridgeport, USA; 2 Internal Medicine, BronxCare Health System, Bronx, USA; 3 Internal Medicine, St. Vincent Medical Center, Bridgeport, USA; 4 Medicine, Lahore Medical and Dental College, Lahore, PAK

**Keywords:** plasmapheresis, eculizumab, thrombotic thrombocytopenic purpura, thrombotic microangiopathies, hematology, ceftriaxone, pulmonary critical care, ttp, medical icu, drug-induced thrombocytopenia

## Abstract

Thrombotic Thrombocytopenic Purpura (TTP) is a subtype of thrombotic microangiopathy (TMA) resulting in thrombocytopenia, anemia, fever, renal and neurological deficits. Although many drugs have been associated with drug-induced TTP, ceftriaxone has never been reported. Our case reports a patient who was started on ceftriaxone and developed TTP. Peripheral smear showed schistocytes and thrombocytopenia. Surprisingly, antibody formation against the metalloproteinase (ADAMTS13) levels were low-normal. The patient was treated with plasmapheresis and eczulimab, leading to platelet recovery and symptom resolution. TTP is a rare disorder and can be acquired or idiopathic. TTP can be diagnosed with normal ADAMTS13 as well. Further research is required to assess the mechanism by which ceftriaxone causes TTP. Physicians should consider the possibility of TTP in patients with similar presentations following ceftriaxone therapy and use it for timely diagnosis and treatment. Early diagnosis and treatment of ceftriaxone-induced TTP can prevent devastating consequences.

## Introduction

Thrombotic thrombocytopenic purpura (TTP) is an autoimmune disorder mediated by antibody formation against the metalloproteinase (ADAMTS13) responsible for von Willebrand factor (vWF) cleavage. The resulting vWF multimers shear red blood cells, causing organ damage, microangiopathic hemolytic anemia, thrombocytopenia, and renal and neurological involvement [1]. The current standard of care protocols includes treatment with plasma exchange, methylprednisolone, and rituximab with the anti-vWF nanobody caplacizumab, which is growing in importance, especially in relapsing patients [2]. While relatively rare, with an annual incidence of 1-13 per million, early diagnosis is essential for survival. Untreated mortality rates stand at 90%; however, this drops to 10-15% with proper treatment [3]. TTP is characterized by a pentad of symptoms, including microangiopathic hemolytic anemia, thrombocytopenia, fever, and renal and neurological dysfunction [4]. TTP can be confirmed by an ADAMTS13 activity assay of <10%, but normal levels do not exclude TTP as it is a clinical diagnosis [4]. Commonly associated drugs include cyclosporine, clopidogrel, ticlopidine, and interferon-alpha [5]. A widely used third-generation cephalosporin, ceftriaxone, is often used to treat pneumonia, skin infections, abdominal infections, and urinary tract infections. While previous cases have been reported on cefepime, cephalexin, and cefuroxime, no other ceftriaxone-induced TTP has been published [6-7]. We describe a case of TTP in a patient with a urinary tract infection (UTI) requiring plasmapheresis and eculizumab. 

## Case presentation

A 92-year-old female patient with a past medical history of diabetes mellitus, hyperlipidemia, hypertension, diverticulosis, internal hemorrhoids, right eye blindness secondary to cataracts, and dementia presented with complaints of decreased oral intake for the last few weeks. The physical examination was unremarkable. The patient has no medical history of anemia, excessive bleeding, or joint pain. The patient's mental status was baseline but with generalized weakness. The patient does not report any mucocutaneous bleeding. Laboratory investigation was significant for urinalysis showing infection, as shown in Table 1.

**Table 1:** Initial laboratory results

Laboratory Test	Patient Values	Reference values
Hemoglobin	12 g/dl	12-16 g/dl
Mean Corpuscular Volume	95.5 fL	80-96 fL
Platelet Count	192 k/ul	150-400 k/ul
White Blood Cell Count	6.5 k/u	4.8-10.8 k/u
Serum Sodium	138 mEq/L	135-145 mEq/L
Serum Potassium	4.2 mEq/L	3.5-5 mEq/L
Serum Creatinine	1.1 mg/dl	0.5-1.5 mg/dl
Blood Urea Nitrogen, Serum	26 mg/dl	6-20 mg/dl
Serum Calcium	8.7 mg/dl	8.5-10.5 mg/dl
Serum Bicarbonate	27 mEq/L	24-30 mEq/L
Serum Albumin	3.9 g/dl	2.9-4.5 g/dl
Serum Bilirubin Total	2.2 mg/dl	0.2-0.9 mg/dl
Alkaline Phosphatase Serum	80 unit/L	43-160 unit/L
Aspartate Transaminase, Serum	30 unit/L	9-30 unit/L
Alanine Aminotransferase, Serum	14 unit/L	5-40 unit/L
Serum Lipase	35 U/L	<=61 U/L

**Figure 1:**
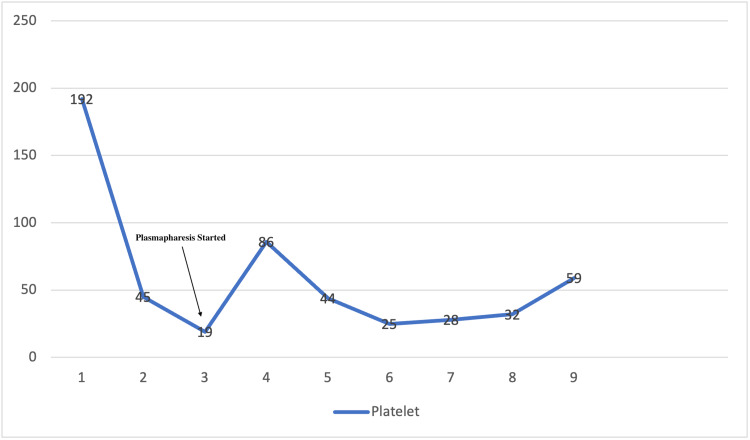
Trend of platelets x-axis shows days in the hospital, y-axis shows platelets (k/ul)

**Figure 2:**
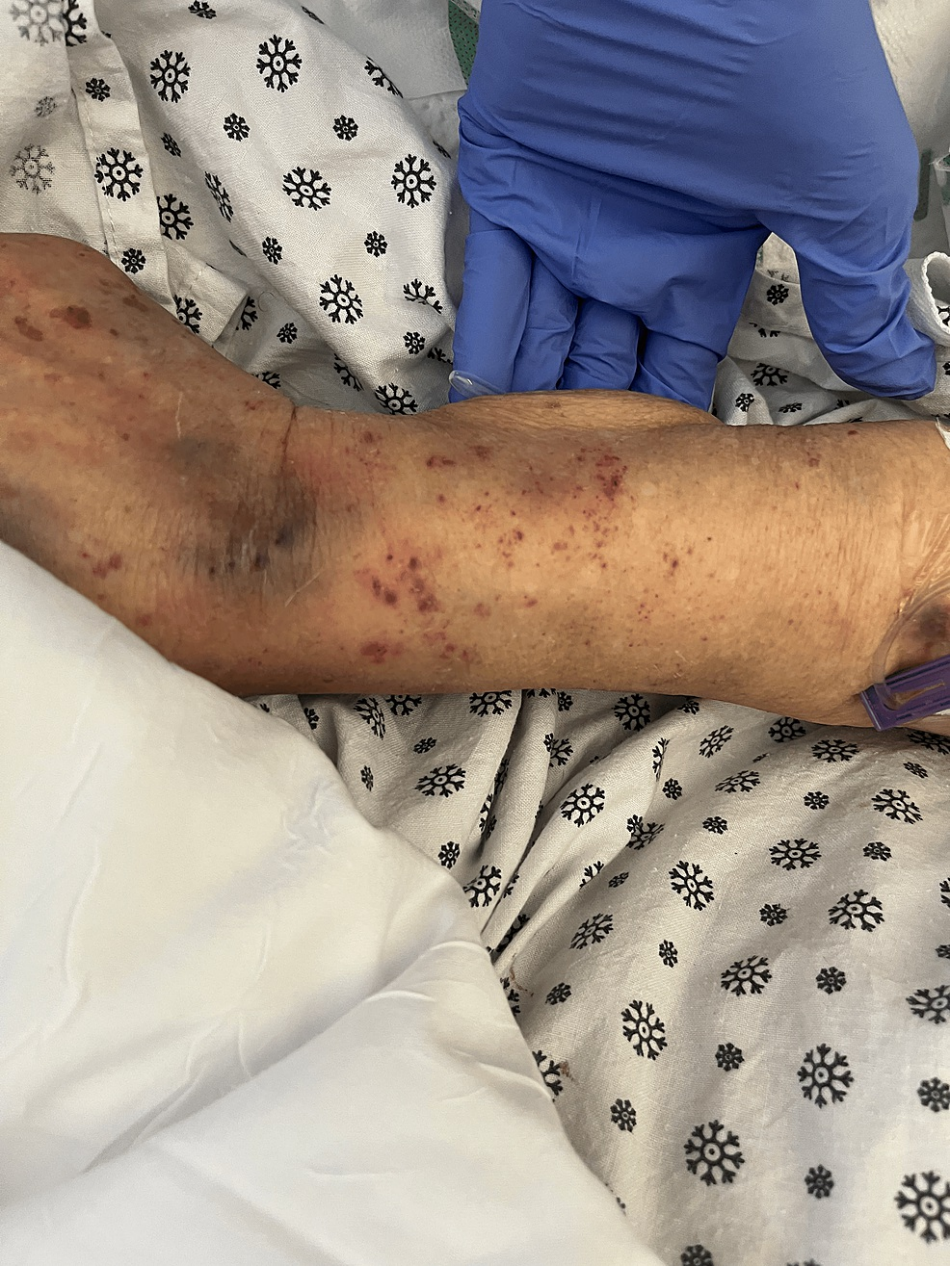
Petechia on the forearm

**Figure 3:**
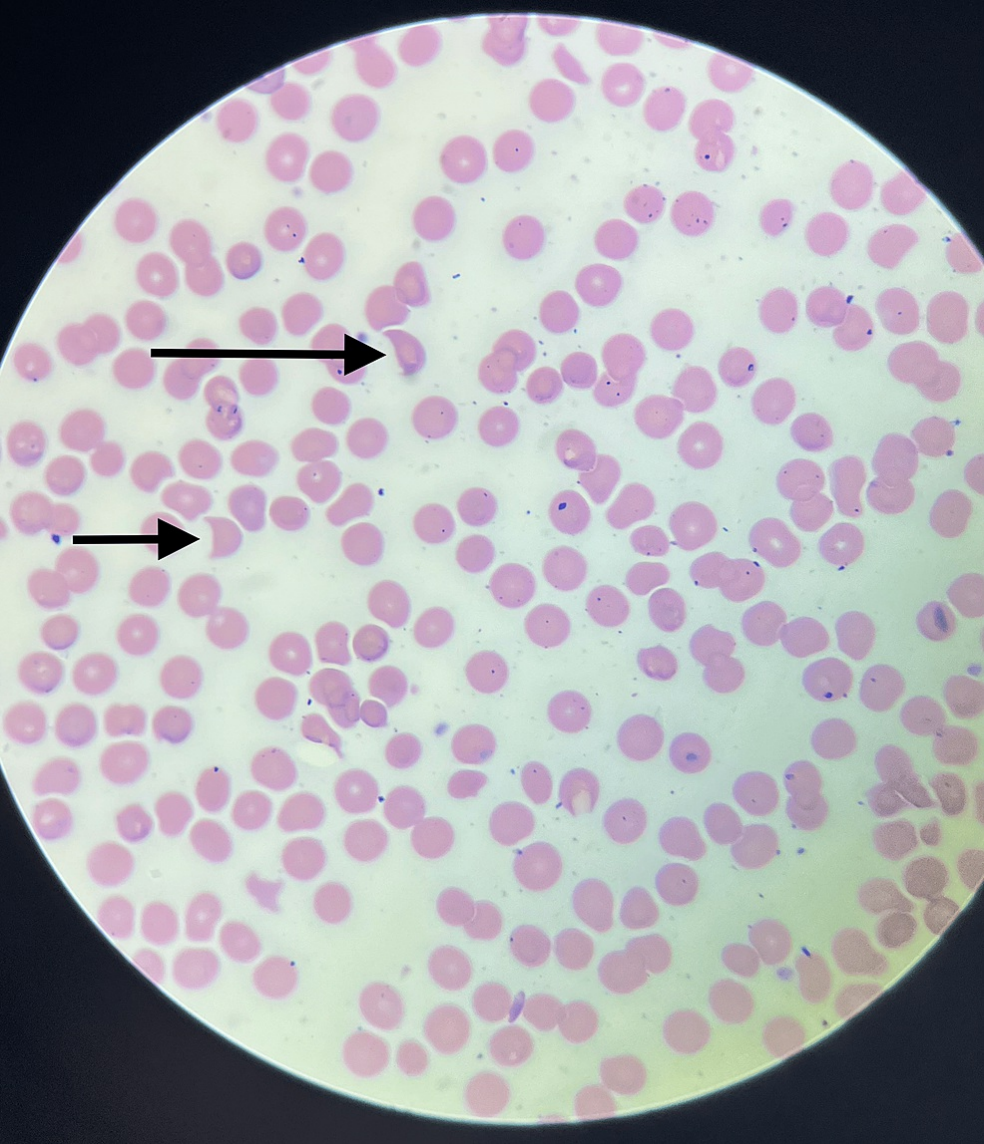
A peripheral smear revealing schistocytes (black arrows) with few platelets

The patient was started on intravenous antibiotic ceftriaxone and intravenous (IV) fluids for concern of UTI. Repeat labs on day two showed a drastic platelet decrease to 45 k/ul. A heparin-induced thrombocytopenia panel was sent. Ultrasound of the abdomen was done, which showed a fatty liver and normal spleen. The platelets kept trending down, as shown in Figure 1. The patient's mental status worsened from baseline with an acute kidney injury with Cr 1.7 mg/dl and thrombocytopenia with a platelet of 45 k/ul on day two. The patient's platelet count; combined hemolysis variable; absence of active cancer; absence of stem-cell or solid-organ transplant; MCV; INR; creatinin (PLASMIC) score was calculated to be 6, which correlates to a 72% risk of severe ADAMTS13 deficiency. The PLASMIC score predicts ADAMTS13 deficiency in suspected thrombotic thrombocytopenic purpura (TTP). During the same time, the patient had a new finding on examination of mild petechiae on the left forearm and peripheral smear showing schistocytes and low platelets, as shown in Figures 2, 3. The patient never received heparin or enoxaparin. Other causes of altered mental status were ruled out, including a CT head, which was negative. Ceftriaxone was stopped under the suspicion of drug-induced TTP. On day two, the patient was transfused platelet and later started on plasmapheresis after consultation with hematology on day three of admission along with a packed red blood cell transfusion. An autoimmune panel, including the ADAMSTS13 antibody, was sent before plasmapheresis. Differential diagnosis included thrombotic microangiopathy (TMA) as well based on lab findings (undetectable haptoglobin, high lactate dehydrogenase (LDH), high indirect bilirubin, anemia, thrombocytopenia), as shown in Table 2. Another suspicion was hemolytic uremic syndrome (HUS), a microangiopathic hemolytic anemia variant likely related to genetic deficiency or antibodies to complement factor. Plasmapheresis was continued for five days, and later, intravenous pulse steroids were started with the plan to transition to oral prednisone if clinical improvement was seen.

**Table 2:** Specific laboratory results for TTP on day 02 LDH: lactate dehydrogenase

Laboratory Test	Patient Values	Reference Values
Serum Haptoglobin	<10.0 mg/dl	30-200 mg/dl
Serum LDH	1729 unit/L	90-285 unit/L
Serum Bilirubin Total	2.3 mg/dl	0.2-0.9 mg/dl
Serum Bilirubin Direct, Conjugated	0.5 mg/dL	0.0-0.3 mg/dL
Hemoglobin	7.1 g/dl	12-16 g/dl
Platelet count	45 k/ul	150-400 k/ul

The patient’s mental status improved on day five. The septic workup was negative to date, and complement or toxin-mediated TMA was also ruled out. The patient's urine culture was negative, and antibiotics were stopped. The patient's creatinine level peaked at 4 mg/dl on day five and then started to trend down. The patient has a mildly elevated coagulation profile, including activated partial thromboplastin time (APTT) in the 30s (normal range 27.2-39.6 s), prothrombin time (PT) 10.5s (normal range 9.9-13.3 s), international normalized ratio (INR) 0.90 (normal range 0.85-1.14), Fibrinogen assay qualitative 383 mg/dl (normal range 182-450 mg/dl) and elevated D-Dimer 5150 ng/ml (normal range <500 ng/ml). The patient had a negative heparin-induced thrombocytopenia (HIIT) panel as well. Plasmapheresis was continued for five days. Surprisingly, ADAMTS13 levels were low-normal, and this makes the case interesting as often drug-induced TTP can have normal ADAMTS13. The plan for kidney biopsy was deferred due to high surgical risk given thrombocytopenia and possible risk of bleeding. The patient also underwent an echocardiogram showing a left ventricle ejection fraction (LVEF) of 76% and otherwise normal findings. The patient was continued on daily plasmapheresis with IV steroids to achieve a platelet count of more than 150,000 k/ul, but the platelet count failed to improve significantly. Later, the patient was transferred to a tertiary care hospital to receive eculizumab. The patient was last seen in the clinic after one week, and the platelet showed improvement.

## Discussion

TTP, a rare thrombotic microangiopathy subtype, has a prevalence rate of 9.5 per 100,000 people with a mortality rate of 95% in untreated cases [8]. TMA has been divided into TTP and hemolytic uremic syndrome (HUS) [9]. TTP was first described by Moschcowitz in 1924; it remains a rare and life-threatening condition [10]. Patients typically present within two weeks of exposure to the offending medicine with severe thrombocytopenia, petechiae, and bleeding [11]. Ceftriaxone is a third-generation cephalosporin used for gonorrhea, otitis media, skin infections, UTIs, bacterial meningitis, and intra-abdominal infections.

In this report, we detail the case of an elderly UTI patient presenting with clinical and laboratory features of TTP following the initiation of ceftriaxone antibiotic. This is the first known case of TTP following ceftriaxone therapy. Our patient displayed the classic symptoms of TTP, including thrombocytopenia, hemolytic anemia, acute kidney injury, and neurological deficits following ceftriaxone initiation. The successful response to IV steroids, plasmapheresis, and eculizumab confirms TTP. Drugs commonly associated with TTP include cyclosporine, tacrolimus, gemcitabine, mitomycin, interferon alpha, and quinine [12]. Ceftriaxone is not yet linked to thrombotic microangiopathies. However, it is commonly associated with drug-induced immune hemolytic anemia (DIIHA) [13][14]. The known adverse reactions to ceftriaxone include injection site reactions, phlebitis, gastrointestinal events, anemia, thrombocytopenia, and biliary pseudolithiasis at higher doses (>2g/day), which may mimic some symptoms of TTP; however, the neurologic involvement and kidney injury seen in this patient would remain unexplained [15].

The patient was introduced to the new drug, ceftriaxone; she received the intravenous (IV) formulation, which resulted in severe thrombocytopenia and mild petechiae. The patient never got any form of heparin. After a thorough visualization of the medication reconciliation, ceftriaxone was promptly recognized and discontinued. Direct visualization of the peripheral smear showed schistocytes. She was immediately transferred to the ICU, and plasmapheresis was started, which resulted in an improvement in laboratory values and symptoms. Newer therapies favor using complement C5 blockers such as eculizumab and ravulizumab for patients with worsening renal function despite supportive care; however, the high cost of these drugs remains a limiting factor in their widespread usage [3, 16-17]. Complement C5 blockers have recently been used in atypical HUS and TTP [16-17]. Our patient was transferred to a tertiary center to receive Eculizumab. 

The patient's PLASMIC score was calculated to be 6, which correlates to a 72 % risk of severe ADAMTS13 deficiency. The PLASMIC score predicts ADAMTS13 deficiency in suspected thrombotic thrombocytopenic purpura (TTP), as shown in Table 3. Our patient ADAMTS13 level came back low-normal. However, there have been incidences when TTP is diagnosed and managed with normal ADAMTS13 levels. The patient's acute kidney injury (AKI) and neurological deficits were not explained by any other diagnosis, as UTI was ruled out, and CT head was normal. There is no direct incidence of drug-induced TTP with normal ADAMTS13 [18-20]. However, clopidogrel-induced TTP has normal ADAMTS13 in 75% of cases [18-20]. Further research is needed to understand the mechanism by which ceftriaxone causes TTP or HUS. Physicians should be aware of this rare complication of this relatively common antibiotic.

**Table 3:** PLASMIC Score System

PLASMIC SCORE SYSTEM
Platelet count of <30,000/μL
Evidence of hemolysis (reticulocyte count >2.5%, elevated indirect bilirubin >2 mg/dL, undetectable to low haptoglobin levels)
Creatinine <2 mg/dL
Mean Corpuscular Volume (MCV) <90 fL,
International Normalized Ratio <1.5
No active cancer or organ/stem cell transplant

## Conclusions

Ceftriaxone-associated TTP has never been reported before. It is a severe but scarce medical condition. It is one of the most extensively used antibiotics. Using this report, physicians should consider the possibility of TTP in patients with similar presentations following ceftriaxone therapy and use it for timely diagnosis and treatment. Early diagnosis and treatment of ceftriaxone-induced TTP can prevent devastating consequences.
